# Phytoplankton responses to temperature increases are constrained by abiotic conditions and community composition

**DOI:** 10.1007/s00442-016-3693-3

**Published:** 2016-08-04

**Authors:** Maren Striebel, Stefanie Schabhüttl, Dorothee Hodapp, Peter Hingsamer, Helmut Hillebrand

**Affiliations:** 1 Institute for Chemistry and Biology of the Marine Environment, University of Oldenburg, Schleusenstrasse 1, 26382 Wilhelmshaven, Germany; 2 Institute of Hydrobiology and Aquatic Ecosystem Management, University of Natural Resources and Life Sciences, Max Emanuel-Strasse 17, 1180 Vienna, Austria; 3 WasserCluster Lunz, Dr. Carl Kupelwieser Promenade 5, 3293 Lunz am See, Austria; 4 Department of Organismic Biology, University of Salzburg, Hellbrunnerstrasse 34, 5020 Salzburg, Austria

**Keywords:** Phytoplankton, Disturbance, Origin effects, Diversity, Nutrients

## Abstract

Effects of temperature changes on phytoplankton communities seem to be highly context-specific, but few studies have analyzed whether this context specificity depends on differences in the abiotic conditions or in species composition between studies. We present an experiment that allows disentangling the contribution of abiotic and biotic differences in shaping the response to two aspects of temperature change: permanent increase of mean temperature versus pulse disturbance in form of a heat wave. We used natural communities from six different sites of a floodplain system as well as artificially mixed communities from laboratory cultures and grew both, artificial and natural communities, in water from the six different floodplain lakes (sites). All 12 contexts (2 communities × 6 sites) were first exposed to three different temperature levels (12, 18, 24 °C, respectively) and afterward to temperature pulses (4 °C increase for 7 h day^−1^). Temperature-dependent changes in biomass and community composition depended on the initial composition of phytoplankton communities. Abiotic conditions had a major effect on biomass of phytoplankton communities exposed to different temperature conditions, however, the effect of biotic and abiotic conditions together was even more pronounced. Additionally, phytoplankton community responses to pulse temperature effects depended on the warming history. By disentangling abiotic and biotic effects, our study shows that temperature-dependent effects on phytoplankton communities depend on both, biotic and abiotic constraints.

**Electronic supplementary material:**

The online version of this article (doi:10.1007/s00442-016-3693-3) contains supplementary material, which is available to authorized users.

## Introduction

An increasing number of studies focus on how phytoplankton primary productivity and species composition are influenced by water temperature, triggered by an interest to understand how global warming affects ecosystem processes and properties (Gerten and Adrian 2002; Carvalho and Kirika 2003; Tadonléké 2010; Yvon-Durocher et al. 2010, 2011; Boyce et al. 2010; De Senerpont Domis et al. 2013; Lewandowska et al. 2014; Cross et al. 2015). The results of these studies, however, differ in that temperature increase can have positive, no, or negative effects on phytoplankton biomass: a long-term temperature increase of 1 °C increased primary production in lakes (Gerten and Adrian 2002), whereas an increase of similar magnitude in sea surface temperature decreased marine phytoplankton biomass (Boyce et al. (2010). A short-term temperature increase of 4 °C resulted in lower phytoplankton biomass (Yvon-Durocher et al. 2010, 2011), while another experiment, establishing a 3 °C temperature increase, showed minor effects of temperature on phytoplankton biomass (Moss et al. 2003).

Given the fact that temperature has fundamental and well understood consequences for metabolism and growth of organisms (Brown et al. 2004) as well as the strength of trophic interactions (Hillebrand et al. 2009), the question remains what is causing this divergence: Here, we use a unique experimental setup to analyze three potential sources of divergence: the abiotic conditions between sites, the species composition of the warmed community (including its history of disturbance events) and the nature of the temperature treatment between pulse and press (permanent increase) disturbance.

The first two of these sources of variation (abiotic conditions and species composition) are strongly interlinked: differences in abiotic conditions might alter temperature effects by direct interactions, e.g., between nutrient availability and temperature effects, as well as by indirect effects mediated by differences in species composition.

Direct effects between temperature and nutrient conditions [as shown for single algal species by Rhee and Gotham (1981)] have been found in numerous empirical studies: depending on nutrient availability the maximal photosynthetic rates per chlorophyll *a* concentrations showed positive, none or negative temperature effects (Tadonléké 2010). Another study showed that warming had positive effects on phytoplankton biomass but was strongly negatively affected by decreasing nutrient flux at higher temperatures (Lewandowska et al. 2014). Furthermore, temperature effects on biomass can also be constrained by altered zooplankton grazing and nutrient availability in combination with increasing temperature (Carvalho and Kirika 2003). Temperature effects also differ for communities of different composition as different algal species and groups differ in their temperature optima (Eppley 1972; Goldman 1977; Thomas et al. 2012) and thus, warming will affect their growth and competitive dominance differently: diatoms tend to be abundant in the well mixed periods of spring and autumn in temperate lakes (Lund 1965) as they tend to benefit from greater mixing (Moss and Balls 1989; Reynolds 1997). Green algae and cyanobacteria have higher optimum temperatures (Seip and Reynolds 1995; Staehr and Sand-Jensen 2006) and tend to benefit from high nutrient concentrations (Jensen et al. 1994; Moss et al. 2003).

 The environmental conditions (and their variability) a community is exposed to determine niche availability and thus the type of functional groups that fill these. Whereas functional group composition tends to be deterministic, species composition within these functional groups often arises stochastically from the history of species arrival (Fukami et al. 2005 and citations therein) and the regional species pool present. In systems undergoing frequent disturbances—such as the floodplain systems investigated here (see below)—the elapsed time since the last disturbance (time for recovery) is important for the assembly and composition of the communities. Floodplain systems are ideal model systems to study how effects of environmental change on ecosystem functioning and biodiversity depend on community assembly due to their pronounced spatial heterogeneity and because changes in abiotic and biotic conditions occur much faster than in most other ecosystems (Tockner et al. 2010). These systems exhibit, among other characteristics, a broad range of nutrient conditions and assembly histories across small spatial scales depending on their connectivity to the river and between each other. The exchange of matter and organisms among habitats of different age and productivity is often pulsed by river discharge and thereby related to abrupt temperature changes (Hein et al. 2004). Therefore, we used the existing variation between floodplain lakes in abiotic conditions and species composition to test for the contingencies of temperature effects in freshwater systems.

To disentangle the compositional and abiotic differences between the lakes (sites), we conducted an experiment allowing for the first time to disentangle direct from indirect contingencies: We imposed the same temperature treatments on communities from six different sites (different abiotic conditions and composition) and on the same artificial community inoculated in water from the same six sites (different abiotic conditions only). Thereby, we are able to disentangle the effects only due to conditions from the effects of the presence/absence of certain species, as community composition determines the traits constraining productivity at different temperatures as well as how environmental conditions affect community productivity (Hillebrand and Matthiessen 2009).

The third source of variation in temperature effects on phytoplankton reflects that temperature increases can comprise aspects of press and pulse disturbances. The terms ‘press’ and ‘pulse’ disturbance have been defined by Bender et al. (1984) and Glasby and Underwood (1996): A pulse disturbance is considered as short-term disturbance that can cause a sudden change in species numbers with likely recovery after the disturbance. A press disturbance is considered as a continuous disturbance along with a permanent change in species abundance and density. Temperature is expected to increase in mean temperatures (press disturbance) and also extreme temperatures (pulse disturbance, e.g., in the form of heat waves) are supposed to increase in frequency and intensity (IPCC 2007). Changes in mean temperature as well as heat waves can have profound effects on phytoplankton communities and thus on ecosystem functioning which may be independent or interactive (synergistic, antagonistic) (Petchey et al. 1999; Adrian et al. 1999; Hansson et al. 2013). To separate the responses of phytoplankton communities to press and pulse temperature effects, we added heat waves to systems with different warming history: We separated the different types of temperature changes by exposing the phytoplankton communities to three different temperature conditions (12, 18, 24 °C) in a first phase (permanently increased temperature, press disturbance,) and afterward exposed them to short-term temperature changes (pulsed heat waves +4 °C increase) in a second phase.

The aim of this study is to analyze potential mechanisms leading to divergent responses to temperature change. The factorial design of the study [3 temperature levels × 6 sites of origin × 2 community types (artificial vs. natural)] is combined with a two-stage application of temperature, pressed disturbance for 14 days and a heat wave (pulse disturbance) of plus 4 °C for 7 h on top of the temperature of the first phase, to test the following hypotheses:

- (H1) Temperature-dependent changes in biomass and community composition depend on the initial composition of a community (composition is determined by the different sites of origin including their disturbance histories).

This hypothesis will be investigated by comparing phytoplankton communities from six sites, which differ in composition. However, since the sites also differed in temperature, disturbance history, and chemical parameters, the difference between sites of origin includes abiotic and biotic differences. To separate these, we replaced the natural communities by one artificially assembled community (removing the biotic factor), leaving only the abiotic differences between sites. This allows testing the next hypothesis:

- (H2) Abiotic conditions have an effect on the response to different temperature conditions of phytoplankton communities. This will be investigated by comparing the effect of changes in temperature on natural and artificial communities under the same experimental conditions.

These hypotheses will be tested for both the press and the pulse disturbance phase of our experiment, which additionally allows addressing our third hypothesis:

- (H3) Phytoplankton community responses (composition and biomass) to pulse temperature effects depend on the warming history.

## Materials and methods

### Experimental setup

We performed laboratory experiments with natural as well as with artificially assembled phytoplankton communities. The natural communities originated from six different sites of the Danube river floodplains near Vienna, Austria: Danube River (site 1) and so-called Eberschüttwasser (site 2), Hanselgrund (site 3), Kühwörther Wasser (site 4), Schwarzes Loch (site 5), and Schönauer Traverse (site 6). Previous studies (Hein et al. 2004; Preiner et al. 2008; Bondar-Kunze et al. 2009) showed that there is a relationship between age (community development), the frequency of disturbance and productivity, and thus the composition of primary producers in this particular river floodplain system. We therefore chose the six sites for our experiment along a disturbance/productivity gradient, with the six phytoplankton communities sampled differing in taxonomic group as well as in species composition, reflecting different nutrient concentrations and water temperatures at the sites of origin (see Table 1). The samples were collected in spring 2010; the last perturbation for all sites was in July 2009, moderate exchange (water level +0.5 m) for Site 6 occurred in March 2010. The sampled water (including the phytoplankton communities) was pre-filtered using a 100 µm mesh screen to remove zooplankton grazers and stored dark and cold while transported to the lab immediately (within few hours). This water from the six sites including the natural phytoplankton communities from the sites was used to set up the ‘natural communities’ treatments.

**Table 1:**
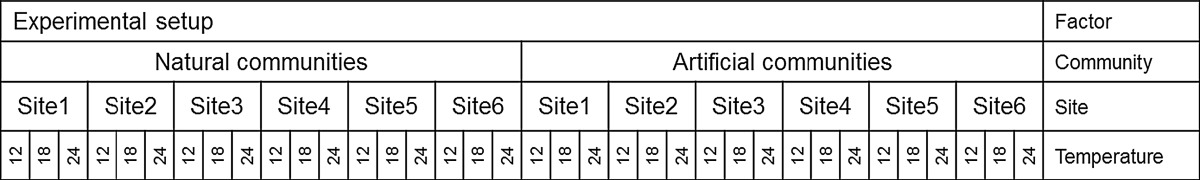
Scheme of the experimental setup including the factors used for analyses: two community types (artificial vs. natural), six sites of origin, and three temperature levels

All treatments were replicated three times resulting in a total of 108 units. The factor ‘Time’ describes the phase (I or II—press or pulse disturbance) of the experiment

Additionally, water was taken from these six sites, filtered (using pre-combusted GF/F filters, Whatman) and autoclaved to eliminate organisms. These pre-treated media from the six sampling sites were used to incubate an artificially assembled community consisting of 12 phytoplankton species from three taxonomic groups: *Chlorophyceae* (green algae), *Cyanophyceae* (cyanobacteria), *Bacillariophyceae* (diatoms) (see also Online Resource 1).The respective cultures were obtained from algal culture collections (SAG Göttingen and CCALA Trebon) and pre-cultured prior to the experiment for months at 18 °C in sterile WC medium according to Guillard and Lorenzen (1972).

The experiment was divided into two phases: experimental phase I—incubation at permanent increased temperatures for 2 weeks, and experimental phase II—pulse temperature exposure for one additional week. For the first phase of the experiment, three constant temperature levels (12, 18, and 24 °C) were established; natural and artificially assembled communities were grown for 14 days to adapt to these temperatures (press disturbance). These temperatures were chosen based on the temperatures at different sites during initial sampling ranging from 12 to 18 °C (see Table 2 showing initial conditions at the different sites). In the second phase, water temperature was increased by 4 °C over a period of 7 h per day to reach 16, 22, and 28 °C, respectively. These temperature peaks were repeated daily for 7 days to simulate strong daily temperature variation (pulse disturbance) in sensitive (small and shallow) water bodies.

**Table 2:** Initial conditions at different sites of origin

Site	Temperature (°C)	Total P (µg P L^−1^)	Chlorophyll *a* (µg L^−1^)	Taxon richness	POP (µg P L^−1^)	POC (mg C L^−1^)	C:P, molar
1—Danube River	12.82	21.97	15.11	15	14.0	2.1	391
2—Eberschüttwasser	15.30	5.26	2.87	30	3.0	0.8	709
3—Hanselgrund	18.52	22.99	3.27	16	11.8	1.0	217
4—Kühwörther Wasser	17.41	9.30	5.97	29	4.9	1.2	606
5—Schwarzes Loch	14.69	51.40	7.34	6	43.0	2.1	127
6—Schönauer Traverse	16.19	14.50	3.55	24	10.9	1.5	356

Cell culture flasks (250 mL) were used as experimental units and were shaken twice a day to keep the algae in suspension. All communities were exposed to a light:dark cycle of 16:8 h at a light intensity of about 100 µmol photons m^−2^ s^−1^ (PAR spectrum; measured in water in the culture flasks); the position of the culture flasks was changed randomly during the experiment to assure equal light conditions for all treatments and to avoid any position effects. The experiment was conducted with an exchange of 10 % day^−1^ (of a total sample volume of 200 mL each). The medium used for the daily exchange was pre-filtered and autoclaved water from each of the sites the communities were sampled from (natural communities) or incubated in (artificial communities), respectively. Light intensity and water temperature were controlled by continuous data logging (HOBO Data Loggers, Onset Computer Corporation). Incubation of the natural communities started off at their natural biomass concentrations of the respective site and sampling day. Artificial communities were started with even proportions of the 12 species used and all treatments were incubated with an equal amount of initial biomass (based on chlorophyll *a* concentrations). Each treatment was replicated three times. At the end of experimental phase I, 50 % of each of the samples was used for analysis and the culture flasks were refilled with fresh medium (as described above) up to the initial sample volume (200 mL). This way, the experimental setup was the same as in a previous laboratory experiment described in Schabhüttl et al. (2013).

### Sampling and analysis

As a proxy for biomass, chlorophyll *a* concentrations were measured fluorometrically (Fluorometer and PhytoPAM, Walz) and samples for particulate organic carbon (POC) and particulate organic phosphorus (POP) analysis were filtered onto pre-combusted and acid-washed glass-fiber filters (Whatman GF/C) at the start of the experiment, after experimental phase I (permanent temperature increase), and again after experimental phase II (temperature pulses). POC was measured by infrared spectrometry (C-Mat 500, Ströhlein). Total phosphorus (TP) and particulate organic phosphorus (POP) concentrations were measured by molybdate reaction after sulfuric acid digestion (Wetzel and Likens 2003). To determine cell numbers and algal biovolume, community samples were taken at the beginning of the experiment as well as at the end of experimental phase I and II, respectively. Samples were fixed with Lugol’s solution (1 % final concentration) and taxonomic composition was determined using an inverted microscope (Utermöhl 1958). Taxon-specific cell volumes were calculated by approximation to simple geometrical shapes (Hillebrand et al. 1999). Community biovolume was calculated as the product of single cell volumes with the corresponding cell densities derived from Utermöhl counting.

### Data analysis

For graphs and statistical analyses R 3.2.0 (R Development Core Team 2011) was used. We tested the natural communities (after press disturbance) for difference in the biomass response (log-transformed chlorophyll *a* concentrations) to temperature between the sites of origin (ANOVA for ‘site’ and ‘temperature’), where a significant interaction between site and temperature indicates differences due to biotic and abiotic conditions. Comparing the effects of abiotic conditions (temperature and site of artificial communities) with the interactive effect of abiotic and biotic conditions (temperature and site of natural communities) will support these results (ANOVA with natural and artificial communities for ‘temperature’, ‘site’, and ‘community’ after press disturbance), where a significant interaction between site and temperature supports H1. Redoing this analysis for the artificial communities tested the importance of the abiotic difference alone (H2): if the interaction between site and temperature remains significant for artificial communities, then abiotic differences between sites suffice to create different temperature effects. If not, then the compositional differences alone drive differential temperature effects. For ANOVAs, we calculated the Eta-Squared measure of effect size (Tabachnick and Fidell 2013; Thompson 2006; Brown 2008).

We used a repeated measures (rm-)ANOVA with log-transformed chlorophyll *a* concentrations as proxy for phytoplankton biomass to test the whole dataset for the effect of community composition (‘site’) on temperature-(‘temperature’) dependent changes in phytoplankton biomass (H1), the effect of abiotic vs biotic conditions (‘community’) on changes in phytoplankton biomass (H2), and whether the phytoplankton community response to pulsed temperature (‘time’) effects depended on warming history (H3). When analyzing the temperature effect on the natural communities after the pulsed temperature disturbance treatment (rm-ANOVA for natural communities), a significant time × temperature interaction supports H3 that warming history alters the response to a heat wave (pulsed temperature disturbance). Additionally, the log response ratio was calculated as the log ratio of chlorophyll *a* concentrations of pulsed disturbance treatments and pressed disturbance treatments [LRR = ln(chlorophyll *a* pulse/chlorophyll *a* press)]. For rmANOVAS we calculated the generalized Eta-Squared measure of effect size (Bakeman 2005).

To further investigate the effects of temperature, abiotic conditions and pulse temperature disturbance on changes in phytoplankton community composition, we calculated an algal response factor on a taxonomic group level (Sarnelle 1992). This response factor is calculated as the log response ratio for the biovolume fraction of one taxonomic group within the community at a point in time, i.e., after permanently increased temperature, divided by the group’s biovolume fraction at an earlier point in time, i.e., relative biovolume at the beginning of the experiment. A positive algal response factor indicates a relative increase in biovolume of one taxonomic group, a negative response factor a decrease. The algal response factor was used to determine the temperature-dependent response in community composition of natural and artificial communities and the effect of press and pulse temperature disturbance: differences between the algal response factors for the different taxonomic groups of natural and artificial communities indicate effects of origin (community composition at the site of origin) (H1) while differences between the pressed and pulsed disturbance would indicate that warming history alters the community response to a heat wave (pulsed temperature disturbance) (H3).

For natural communities, we calculated Bray–Curtis dissimilarity in phytoplankton community composition (using relative biovolume) between the different sites of origin (‘site’) using the R package “vegan” (Oksanen et al. 2012). Dissimilarity was calculated based on taxon composition as well as on functional group composition for all three temperature levels at the beginning of the experiment, after pulse and after press disturbance.

## Results

### Temperature-dependent changes in phytoplankton biomass

**Fig. 1:**
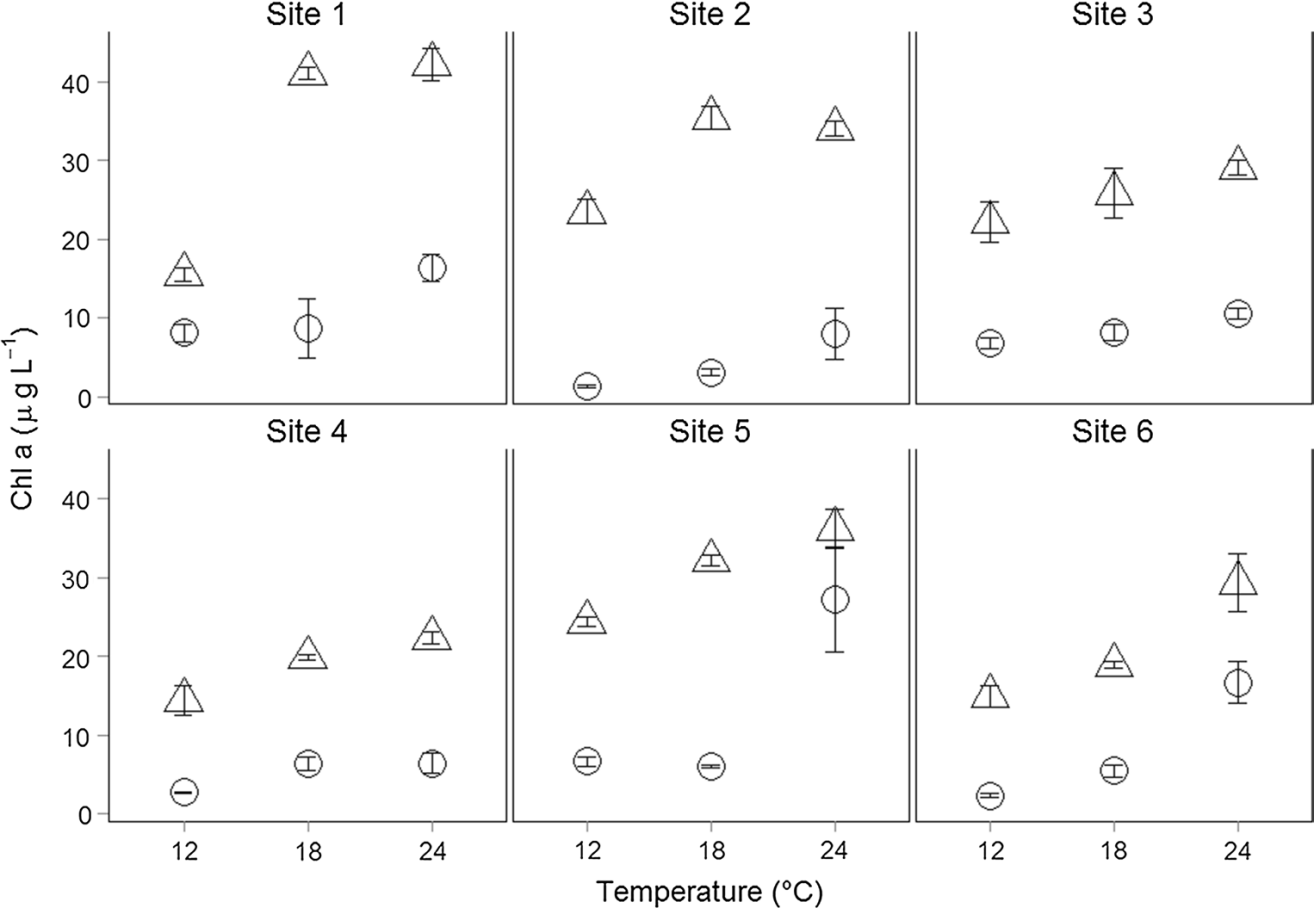
Phytoplankton biomass after press disturbance. Chlorophyll *a* concentrations (µg L^−1^) at three temperature levels (12, 18, and 24 °C) for natural (*open dots*) and artificial (*open triangles*) communities after press disturbance (phase I). Data are shown as mean (± SE). *Site 1* Danube River, *Site 2* Eberschütt Wasser, *Site 3* Hanselgrund, *Site 4* Kühworter Traverse, *Site 5* Schwarzes Loch, *Site 6* Schönauer Traverse

In the first phase of the experiment (press disturbance), the biomass of artificial and natural phytoplankton communities increased with higher temperature (Fig. 1). Phytoplankton biomass (here as chlorophyll *a* concentrations) was affected by temperature (significant main effect ‘temperature’, rm-ANOVA and effect size of 0.515, Table 3) and was significantly different between community types (artificial > natural, significant main effect and effect size of 0.856, Table 3) and sites of origin (significant main effect of site and effect size of 0.631, Table 3). As predicted in our hypotheses, the biomass response to temperature differed between community types (natural vs artificial) and sites of origin (all two-way and three-way interactions significant, Table 3).

**Table 3:** Repeated measures ANOVA with log-transformed chlorophyll *a* concentrations including all data

Effect	DFn	*F*	*P*	Effect size
Temperature	2	49.6	<0.0001	0.515
Site	5	31.9	<0.0001	0.631
Community	1	554.4	<0.0001	0.856
Time	1	8.3	0.0052	0.026
Temperature:site	10	3.0	0.0030	0.245
Temperature:community	2	6.1	0.0036	0.115
Site:community	5	15.9	<0.0001	0.460
Temperature:time	2	74.4	<0.0001	0.322
Site:time	5	4.7	0.0008	0.070
Community:time	1	143.0	<0.0001	0.313
Temperature:site:community	10	3.2	0.0017	0.258
Temperature:site:time	10	4.6	<0.0001	0.129
Temperature:community:time	2	35.9	<0.0001	0.187
Site:community:time	5	5.8	0.0001	0.085
Temperature:site:community:time	10	7.0	<0.0001	0.182

Error DFd were 72. Effect size is the generalized Eta-Squared measure of effect size

For natural communities, a significant interaction of temperature × site (ANOVA with natural communities after press disturbance, Table 4) reflected the variable responses to the temperature treatment. The comparison between natural (combined effect of abiotic and biotic conditions) and artificial communities (effect of abiotic conditions) resulted in a significant temperature × site × community interaction supporting H1, assuming that temperature-dependent changes in biomass depend on the initial composition of a community. For artificial communities the temperature × site interaction also remained significant (ANOVA with artificial communities, Table 4), indicating an effect of abiotic conditions (supporting H2).

**Table 4:** Summary of ANOVA’s with log-transformed chlorophyll *a* concentrations of natural and artificial communities after press disturbance

Effect	DFn	*F*	*P*	Effect size
Natural communities: Adj *R* ^2^ 0.81, *F*_17,36_ = 14.1, *P* < 0.0001				
Temperature	2	55.3	<0.0001	0.402
Site	5	18.2	<0.0001	0.330
Temperature:site	10	3.8	0.0015	0.138
Artificial communities: Adj *R* ^2^ 0.88, *F*_17,36_ = 23.2, *P* < 0.0001				
Temperature	2	91.6	<0.0001	0.425
Site	5	30.1	<0.0001	0.349
Temperature:site	10	6.1	<0.0001	0.142
Natural and artificial communities: Adj *R* ^2^ 0.92, *F*_35,72_ = 38.2, *P* < 0.0001				
Temperature	2	101.5	<0.0001	0.144
Site	5	23.5	<0.0001	0.083
Community	1	820.6	<0.0001	0.582
Temperature:site	10	3.8	<0.001	0.027
Temperature:community	2	17.9	<0.0001	0.025
Site:community	5	15.8	<0.0001	0.056
Temperature:site:community	10	4.3	<0.0001	0.031

Effect size is the Eta-Squared measure of effect size

**Fig. 2:**
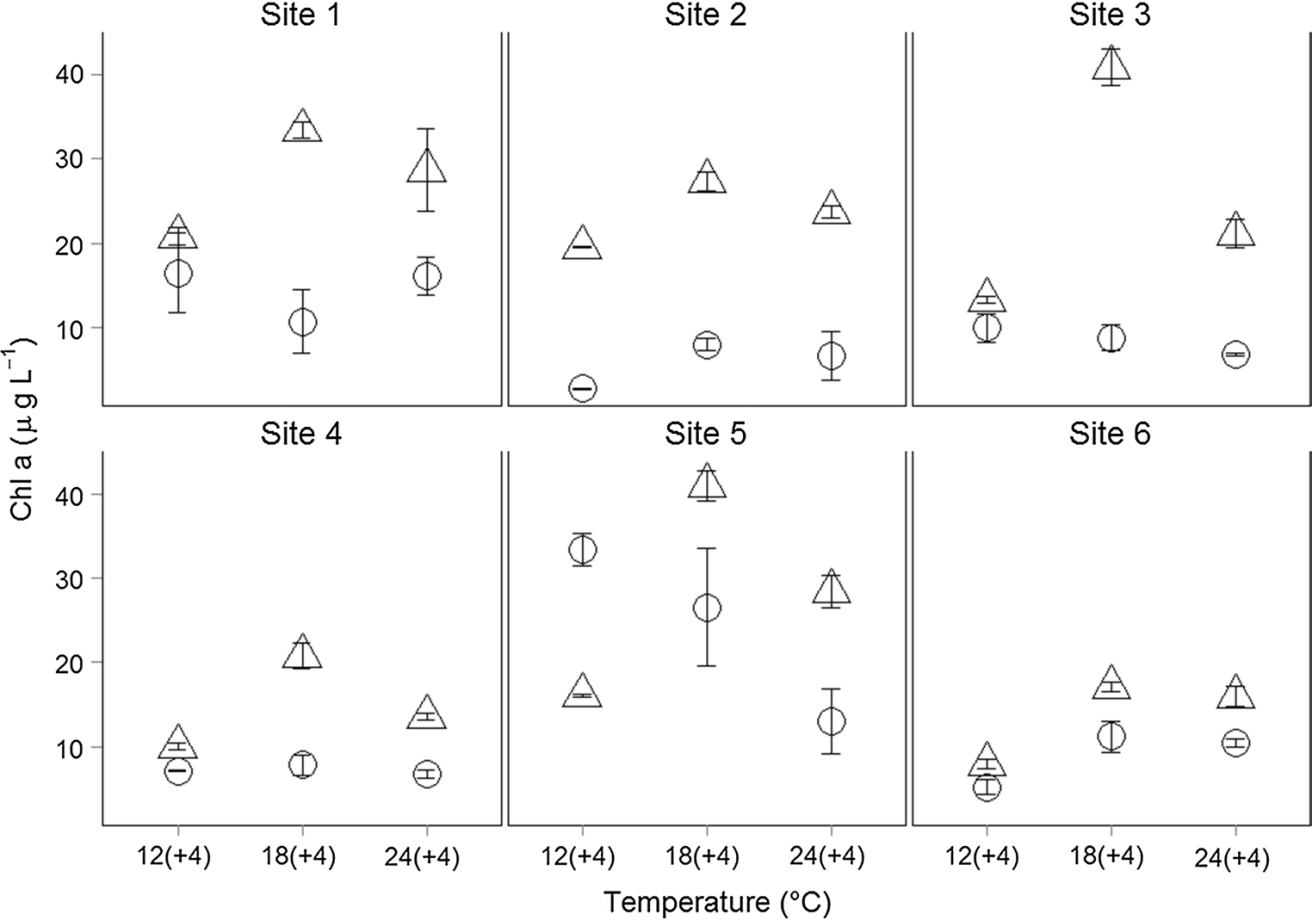
Phytoplankton biomass after pulse disturbance. Chlorophyll *a* concentrations (µg L^−1^) at three temperature levels (12(+4), 18(+4), and 24(+4) °C) for natural (*open dots*) and artificial (*open triangles*) communities after pulse disturbance (phase II). Data are shown as mean (± SE)

After the temperature pulses (phase II of the experiment), phytoplankton biomass in all artificial communities was lowest at 12 °C (+4 °C) while no general trend for temperature-dependent differences in biomass existed in natural communities (Fig. 2, significant community × time and community × temperature × time effects, Table 3). However, pulsed disturbance effects on biomass depended also on site-specific differences (significant interaction temperature × site × time) in natural communities (Fig. 2; Table 5).

**Table 5:** Repeated measures ANOVA with log-transformed chlorophyll *a* concentrations of natural communities

Effect	DFn	*F*	*P*	Effect size
Temperature	2	14.3	<0.0001	0.383
Site	5	19.7	<0.0001	0.682
Time	1	64.8	<0.0001	0.280
Temperature:site	10	3.1	<0.01	0.404
Temperature:time	2	56.7	<0.0001	0.405
Site:time	5	4.9	<0.01	0.127
Temperature:site:time	10	4.8	<0.001	0.225

Error DFd were 36; Effect size is the generalized Eta-Squared measure of effect size

**Fig. 3:**
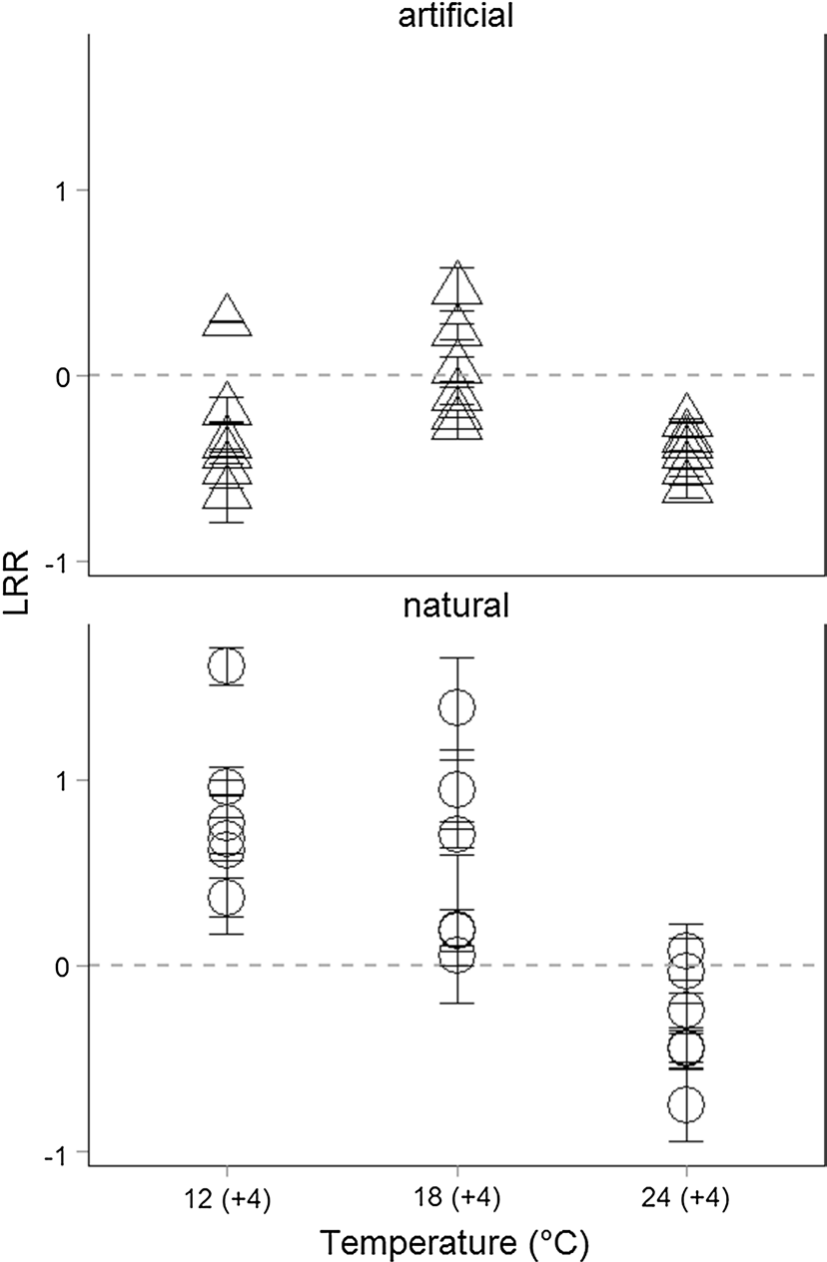
Log response ratio of chlorophyll *a* concentrations. Log response ratio (LRR) calculated as the log ratio of chlorophyll *a* concentrations after pulsed disturbance vs pressed disturbance of natural (*open dots*) and artificial (*open triangles*) communities at different temperatures. Data are shown as mean (± SE) for each site. The *dashed line* indicates no change in chlorophyll *a* concentrations; above the *dashed line* the chlorophyll *a* concentrations were higher after pulse, below the *dashed line* higher after press disturbance

While the absolute biomass of the artificial communities was generally higher than the biomass of natural communities (during both phases of the experiment, Figs. 1, 2), it decreased during the temperature pulses (log response ratios, Fig. 3) in artificial communities at 12 and 18 °C but increased in natural communities at 12 and 18 °C compared to press temperature disturbance. At the highest temperature 24 °C, temperature pulses resulted in a decrease of biomass in both, natural and artificial, communities (supporting H3). Additionally, the significant interaction between ‘temperature’ and ‘community’ on the LRR of chlorophyll *a* concentrations (ANOVA, Table 6) indicates effects of community composition on temperature-dependent changes in chlorophyll *a* concentration due to disturbance (supporting H1).

**Table 6:** ANOVA for LRR of natural and artificial communities

Effect	DFn	*F*	*P*	Effect size
Temperature	2	74.4	<0.0001	0.246
Site	5	4.8	<0.001	0.039
Community	1	143.0	<0.0001	0.236
Temperature:site	10	4.6	<0.01	0.077
Temperature:community	2	36.0	<0.0001	0.119
Site:community	5	5.8	<0.001	0.048
Temperature:site:community	10	7.0	<0.001	0.115

Adj *R* ^2^ 0.82, *F*_35,72_ = 15.2, *P* < 0.0001. Effect size is the Eta-Squared measure of effect size

### Temperature-dependent changes in phytoplankton community composition

**Fig. 4:**
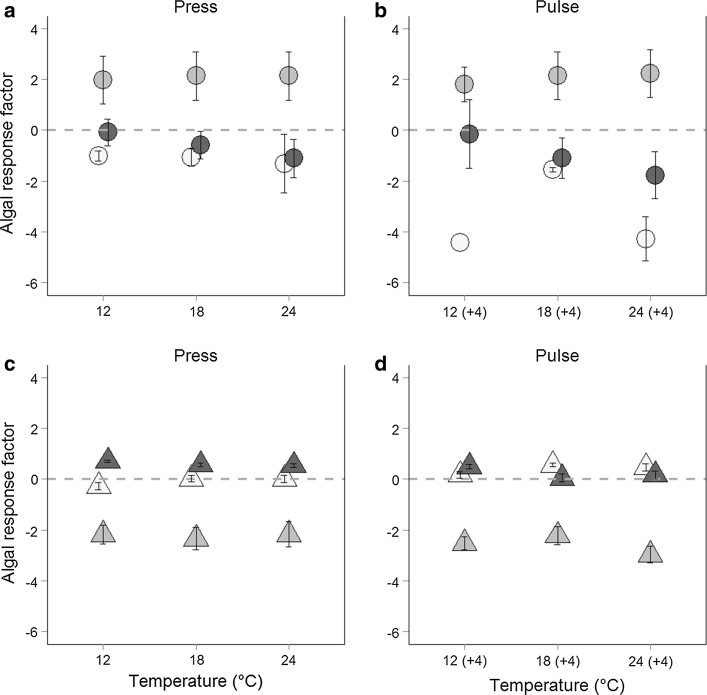
Algal response factor for natural and artificial communities. Algal response factor for natural (*dots*, **a** and **b**) and artificial (*triangles*, **c** and **d**) phytoplankton communities after press disturbance (**a**, **c**) and after pulse disturbance (**b**, **d**). The three most abundant taxonomic groups in all communities, cyanobacteria (*white fill*), diatoms (*light gray fill*), and green algae (*dark gray fill*) are displayed here as means for all sites (± SE). *Symbols* are slightly displaced for better illustration

In natural communities (Fig. 4a, b), diatoms showed a positive response factor, i.e., increased in proportion to total biomass over time, after press temperature disturbance [mean (± SE) response factor across all temperatures = 2.08 (± 0.05)] as well as after pulse temperatures [2.06 (± 0.13)]. Thus, diatoms were able to increase their biovolume proportions during the experiment across all temperature regimes (see also Online Resource 2). By contrast, green algae showed no (response factor close to zero at 12 °C incubation temperature) or negative response factors after press [mean (± SE) response factor across all temperatures = −0.59 (± 0.3)] and pulse (−1.0 (± 0.47)] temperature disturbance; thus their biovolume proportions decreased during the experiment, especially at higher temperatures (Fig. 4). The same is true for cyanobacteria, where the response factor was negative after press [mean (± SE) response factor across all temperatures = −1.13 (± 0.09)] as well as after pulse [− 3.42 (± 0.93)] temperature disturbance.

In artificial communities (Fig. 4c, d; Online Resource 3), in which all three taxonomic groups had started at equal biovolume proportions, diatoms decreased and green algae increased in proportion over time. Diatoms showed negative response factors after press temperature disturbance [mean (± SE) response factor across all temperatures = −2.23 (± 0.06)] as well as after pulse temperatures [− 2.57 (± 0.22)]. Green algae showed positive response factors after press [0.61 (± 0.05)] and pulse [0.24 (± 0.13)] temperature disturbance, while cyanobacteria proportions remained unchanged after press temperature disturbance and were slightly positive after pulse temperatures [0.4 (± 0.12)]. Cyanobacteria had a higher response factor than green algae only after the pulse temperatures and then showed increased relative biovolume compared to green algae and diatoms.

**Fig. 5:**
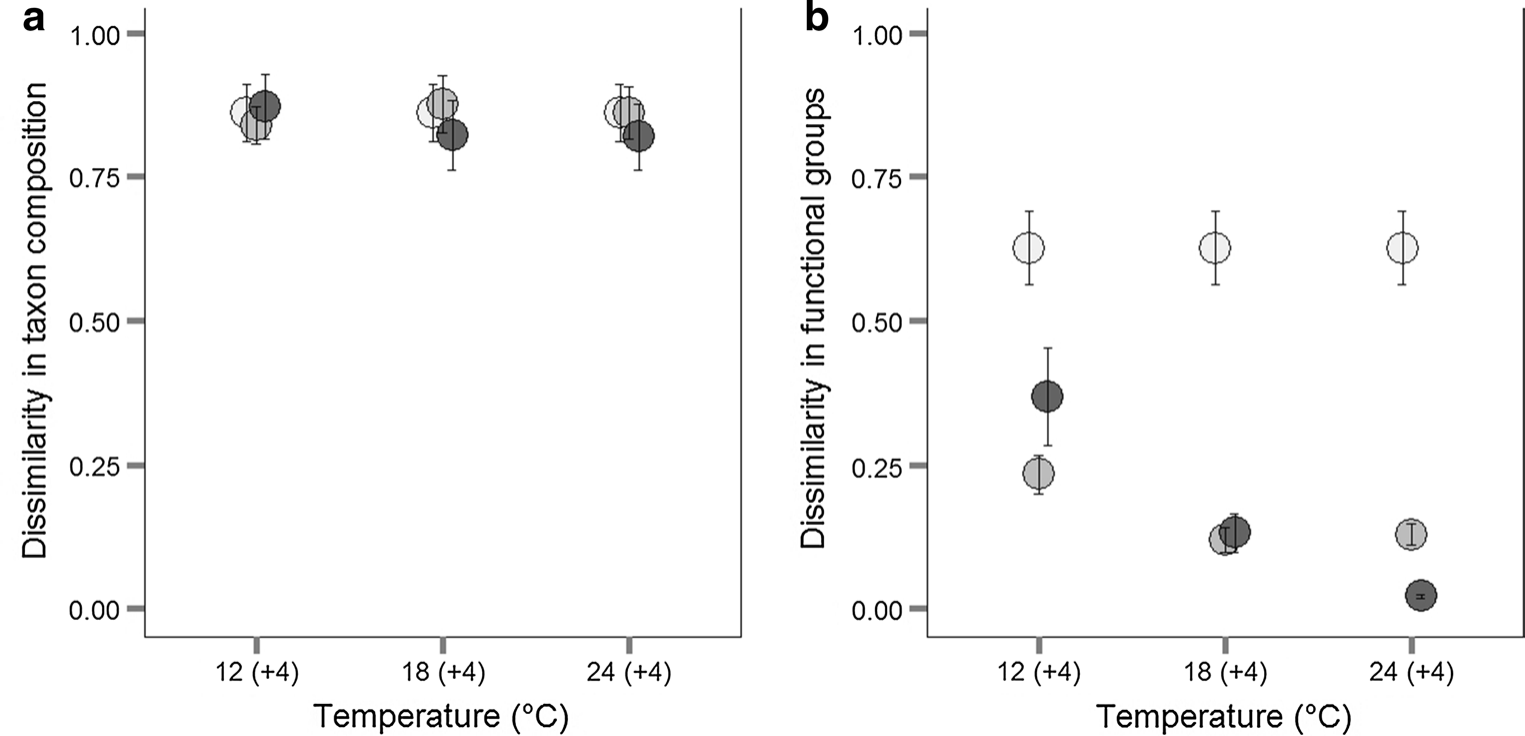
Dissimilarity between sites. Mean (± SE) dissimilarity (Bray–Curtis) between the different sites was calculated based on species composition (**a**) and functional groups composition (**b**) for the most abundant taxonomic groups (diatoms, green algae, cryptophytes, chrysophytes, cyanobacteria, and dinoflagellates). Dissimilarity was calculated for all natural communities at different temperature treatments (12, 18, 24 °C) at the beginning of the experiment (*white fill*), after press temperature exposure (*light gray fill*), and after pulse temperature exposure (*dark gray fill*). *Symbols* are slightly displaced for better illustration

Although all six natural communities had started off at different taxon and functional group composition, all natural communities developed toward diatom-dominated communities (Fig. 5; Online Resource 2). The dissimilarity in terms of functional group composition of the communities (Fig. 5b) was high at the beginning, but significantly lower after press and after pulse temperature disturbance. Thus, the natural communities converged based on functional composition, especially at high temperatures. However, the natural communities remained distinct in taxon composition (Fig. 5a). Additionally, linear regression analyses indicated that the relation between temperature and dissimilarity in terms of functional groups also depended on the type of disturbance treatment: for pulse disturbance the slope was steeper than for press disturbance [press disturbance: *r*^2^ = 0.2, *p* = 0.007, slope = −0.009 (SE = 0.003), *n* = 54; pulse disturbance: *r*^2^ = 0.34, *p* < 0.0001, slope = −0.03 (SE = 0.006), *n* = 54].

## Discussion

### Temperature-dependent changes in phytoplankton biomass and community composition

Temperature-dependent effects on phytoplankton biomass were observed in natural as well as in artificial communities after press and pulse disturbance. Increasing temperature tended to result in a positive effect on phytoplankton biomass in natural (40.2 % of variance explained by temperature, see Table 4) and, even more pronounced, in artificial phytoplankton communities (42.5 % of variance explained by temperature, see Table 4) after press disturbance. After pulse disturbance, however, highest biomass in artificial communities occurred at intermediate (18 °C) temperature while no clear effect of temperature on biomass was observed in natural communities. Such differential effects of warming on phytoplankton productivity have been reported in the literature before. Positive effects of temperature on primary production are often associated with increasing metabolic rates (Yvon-Durocher and Allen 2012) while such positive physiological effects of temperature increases might eventually turn into negative effects as soon as the optimal temperature range is exceeded. This can be caused by cellular stress, due to high membrane fluidity and enhanced degradation rates of proteins and enzymes, and if these effects become significant they can negatively affect primary production (Raven and Geider 1988; Staehr and Sand-Jensen 2006). Optimal temperature ranges depend on the composition of the communities investigated as different species or groups differ in their optimal temperature ranges (Eppley 1972; Goldman 1977; Thomas et al. 2012). Thus, species-specific growth rates dependent on temperature and nutrient conditions and competition results in specific species assemblages. Descamps-Julien and Gonzalez (2005) showed that the biomasses of two competing species under fluctuating temperatures were different than expected from growth experiments on monocultures and temperature fluctuations induced compensatory dynamics resulting in the coexistence of the two species. In this study, we found a clear effect of the site of origin in natural (33.0 % of variance explained by site, see Table 4) as well as in artificial communities (34.9 % of variance explained by site, see Table 4), and thus differences due to abiotic conditions (artificial communities, H2) as well as the combined effect of abiotic and biotic conditions (natural communities). The significant interaction of the site and community when analyzing natural and artificial communities together supports our hypothesis H1 that temperature-dependent changes in biomass depend on the initial composition of a community (biotic conditions).

### Abiotic effects on biomass development and composition of phytoplankton communities

Because all abiotic parameters in our experiment, such as light and nutrient concentration (in the medium), as well as the frequency of medium exchange were exactly the same for the respective natural and artificial communities, the initial community composition remained as the exclusive difference. Thus comparing the results obtained from natural communities with those from the artificial communities, we can conclude that temperature-dependent changes in community composition depend on the initial composition of a community including its disturbance history.

Considering only the abiotic differences between sites (artificial communities only), we still observed an interaction of temperature and site of origin (Fig. 1; Table 4). As for artificial communities, ‘site’ means differences in abiotic conditions; we can conclude that these differences had a major effect on the temperature-dependent effects on biomass, supporting H2. Other studies have also investigated the strongly linked effects of temperatures and changing nutrient conditions (Carvalho and Kirika 2003; Tadonléké 2010; Yvon-Durocher et al. 2010, 2011; Boyce et al. 2010; De Senerpont Domis et al. 2013). Tadonléké (2010) described the combined effects of temperature changes and changes in nutrient conditions based on a long-term observation in a lake and found direct negative effects of warming on primary productivity under phosphorus limitation, while the effects were positive during eutrophic conditions. These studies as well as our results show that temperature effects are strongly linked to other abiotic factors and that these interactions can lead to divergent responses to temperature changes.

Jöhnk et al. (2008) showed that, in natural systems, high temperatures favor cyanobacteria directly through increased growth rates but also indirect by changes in abiotic effects (reduced vertical turbulent mixing, increased light availability). The composition of artificial communities in our experiment, in which the abiotic effects (nutrient concentrations) were the main drivers, developed toward cyanobacteria and green algal-dominated communities compared to natural communities, where the interaction of abiotic and biotic effects resulted in a strong development toward diatom-dominated communities (Fig. 4), also supporting H2. These results also support the conclusions from a previous experiment, comparing the performance of monocultures and mixed communities under different temperature conditions (Schabhüttl et al. 2013), that species interactions, e.g., competitive behavior in communities of various phytoplankton taxa, constitute a key factor for the community development and species composition in phytoplankton communities.

### Warming history effects phytoplankton community response

Different temperature effects on phytoplankton biomass were observed between natural and artificial communities, as artificial communities showed generally higher absolute biomass while natural communities were able to obtain high biomass after pulsed temperature disturbance (Figs. 1, 2, 3). Especially the higher and positive response ratio (LRR) of natural communities to pulse disturbance at 12 and 18 °C (Fig. 3) indicates that the natural communities are better adapted to disturbances than the artificially assembled communities. This was also supported by the significant interactive effects of site, temperature, and time in natural communities (ANOVA, Table 5). These results are congruent with the fact that phytoplankton species are able to acclimate to changes in water temperature within the timeframe of a few generations (Coles and Jones 2000; Staehr and Sand-Jensen 2006). The negative response ratios at 24 °C observed for natural as well as for artificial communities and the temperature-dependent decreasing response ratio of natural communities indicate that the warming history during the experiment (press disturbance at three temperature levels) also affected the phytoplankton community response in terms of biomass. The community composition was also affected by the warming history: the dissimilarity of functional groups decreased more strongly at high temperatures (indicated by a negative slope; Fig. 5) and this decrease was slightly stronger after pulse disturbance than after press disturbance.

While the dissimilarity in functional groups between the different sites (different natural communities) was strongly negatively affected by press and pulse disturbance as well as increasing temperature (mainly diatoms remained in the natural communities), the compositional dissimilarity between the different sites was high at the beginning of the experiment and remained high after press and pulse disturbance. These data corroborate results from experiments with plant communities, which also showed communities’ functional convergence over time alongside taxonomic divergence (Fukami et al. 2005). In general, aquatic and terrestrial studies show that (more frequently or more recently) disturbed communities are more similar than undisturbed communities (Inouye and Tilman 1995; Chase 2003, 2007). A possible explanation might be that under high disturbance lower population densities lead to less intraspecific and interspecific competition while the ability to cope with disturbance becomes more relevant (Grime 1979; Jiang and Patel 2008). Increasing disturbance might cause density reduction or extinction of disturbance-intolerant species that were competitively superior prior to disturbance. The initial communities used in our experiment were sampled in late spring and thus still contained spring-bloom forming species (mainly diatoms) adapted to changing and fluctuating environmental conditions—even if these species were less abundant at the beginning and other, more competitive species, dominated the initial communities. As we reduced the competition for nutrients by exchanging 10 % of the medium per day and at the same time increased the disturbance, we obtained conditions which obviously favored diatoms in our natural communities.

## Conclusions

This experiment confirmed our expectation that temperature-dependent changes in biomass and community composition depend on the initial composition (determined by different sites of origin) of phytoplankton communities. Abiotic conditions had an effect on biomass of phytoplankton communities exposed to different temperature conditions, however, the effect of biotic and abiotic conditions together was even more pronounced. Phytoplankton community responses to pulse temperature effects depended on the warming history concerning the effects on biomass and community composition: taxon composition diverged while functional group composition converged.

## Electronic supplementary material

Below is the link to the electronic supplementary material.

Supplementary material 1 (PDF 3123 kb)
