# Supplementary material for: Phytoplankton responses to temperature increases are constrained by abiotic conditions and community composition
Source: Oecologia. 2016 Aug 4;182(3):815–27. doi: 10.1007/s00442-016-3693-3 (PMC5042995; doi:10.1007/s00442-016-3693-3)
Supplement: Supplementary file 1 — Supplementary material 1 (PDF 3123 kb) [file 442_2016_3693_MOESM1_ESM.pdf]

Phytoplankton responses to temperature increases are constrained by abiotic conditions and community composition

Maren Striebel[1,2], Stefanie Schabhüttl[3], Dorothee Hodapp[1], Peter Hingsamer[4] and Helmut Hillebrand[1]

[1]Institute for Chemistry and Biology of the Marine Environment, University of Oldenburg, Schleusenstrasse 1, 26382 Wilhelmshaven, Germany

[2] Institute of Hydrobiology and Aquatic Ecosystem Management, University of Natural Resources and Life Sciences, Max Emanuel-Strasse 17, 1180 Vienna, Austria

[3] WasserCluster Lunz, Dr. Carl Kupelwieser Promenade 5, 3293 Lunz am See, Austria

[4] Department of Organismic Biology, University of Salzburg, Hellbrunnerstrasse 34, 5020 Salzburg, Austria

Author Contributions:

MS and SS conceived, designed and performed the experiments. MS, DH and PH analyzed the data. MS and HH wrote the manuscript; other authors provided editorial advice.

Electronic Supplemental Material Striebel et al.

Online Resource 1: Summary of phytoplankton species used for the artificially assembled communities

Genus	Species	Group	Origin/Strain number
Chlamydomonas	reinhardtii	*Chlorophyceae*	SAG 11-31
Mougeotia	scalaris	*Chlorophyceae*	SAG 164.80
Scendedesmus	obliquus	*Chlorophyceae*	SAG 276-10
Staurastrum	tetracerum	*Chlorophyceae*	SAG 7.94
Anabaena	cylindrica	*Cyanophyceae*	SAG 1403-2
Chroococcus	minutus	*Cyanophyceae*	SAG 41.79
Leptolyngbya	fragilis	*Cyanophyceae*	CCALA 87 HINDAK 1982/12
Oscillatoria	limnosa	*Cyanophyceae*	SAG 42.87
Fragilaria	crotonensis	*Bacillariophyceae*	SAG 28.96
Navicula	pelliculosa	*Bacillariophyceae*	SAG 1050-3
Nitzschia	palea	*Bacillariophyceae*	SAG 1052-3a
Skeletonema	subsalum	*Bacillariophyceae*	SAG 8.94

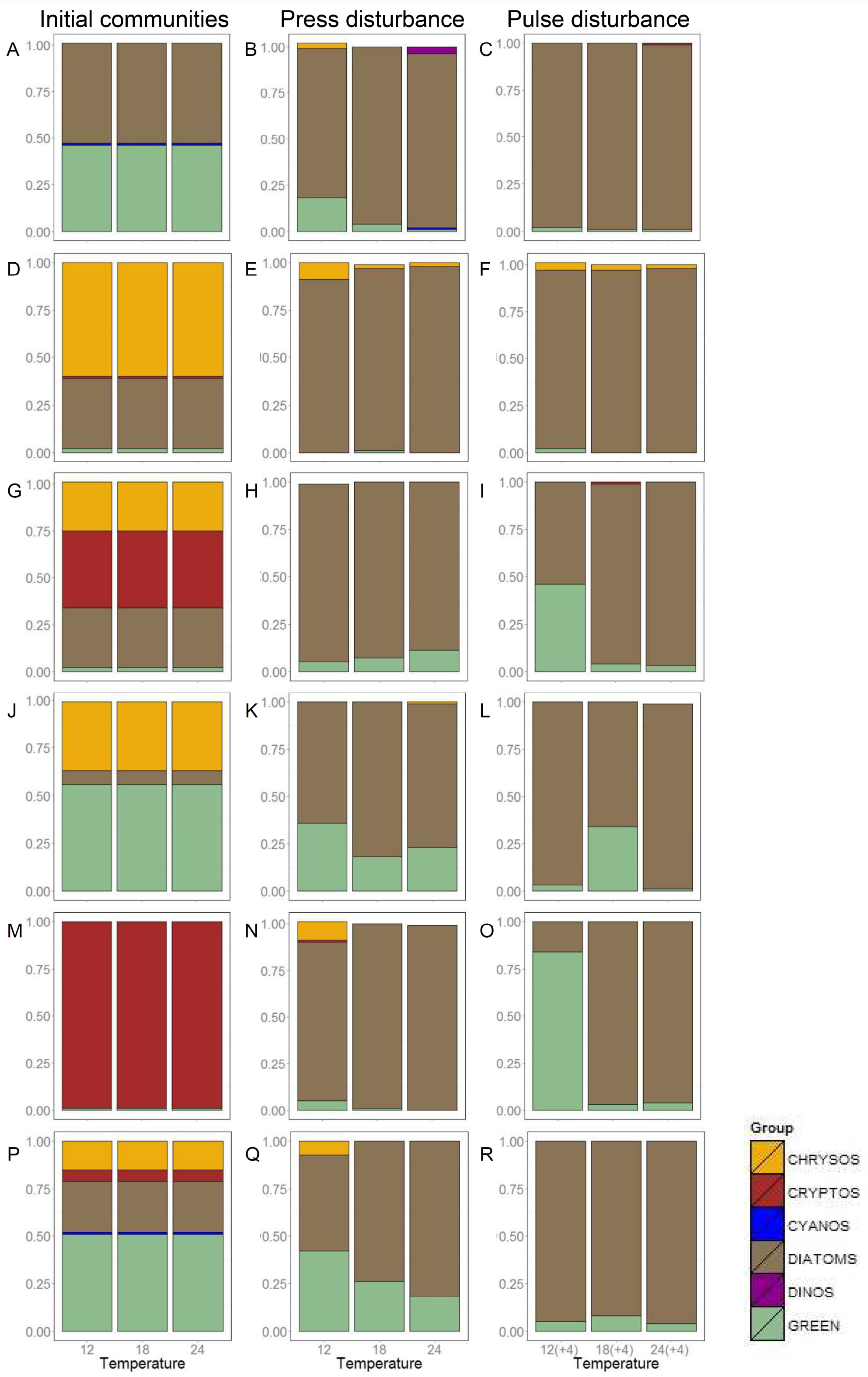

Online Resource 2: Community composition (relative biovolume) of natural communities based on functional groups (Chrysophytes, Cryptophytes, Cyanobacteria, Diatoms, Dinoflagellates, Green algae) for initial communities, communities after press disturbance and after peak disturbance. A-C Site1 (Danube River), D-F Site2 (Eberschütt Wasser). G-I Site3 (Hanselgrund), J-L Site4 (Kühworter Traverse), M-O Site5 (Schwarzes Loch), P-R Site6 (Schönauer Traverse).

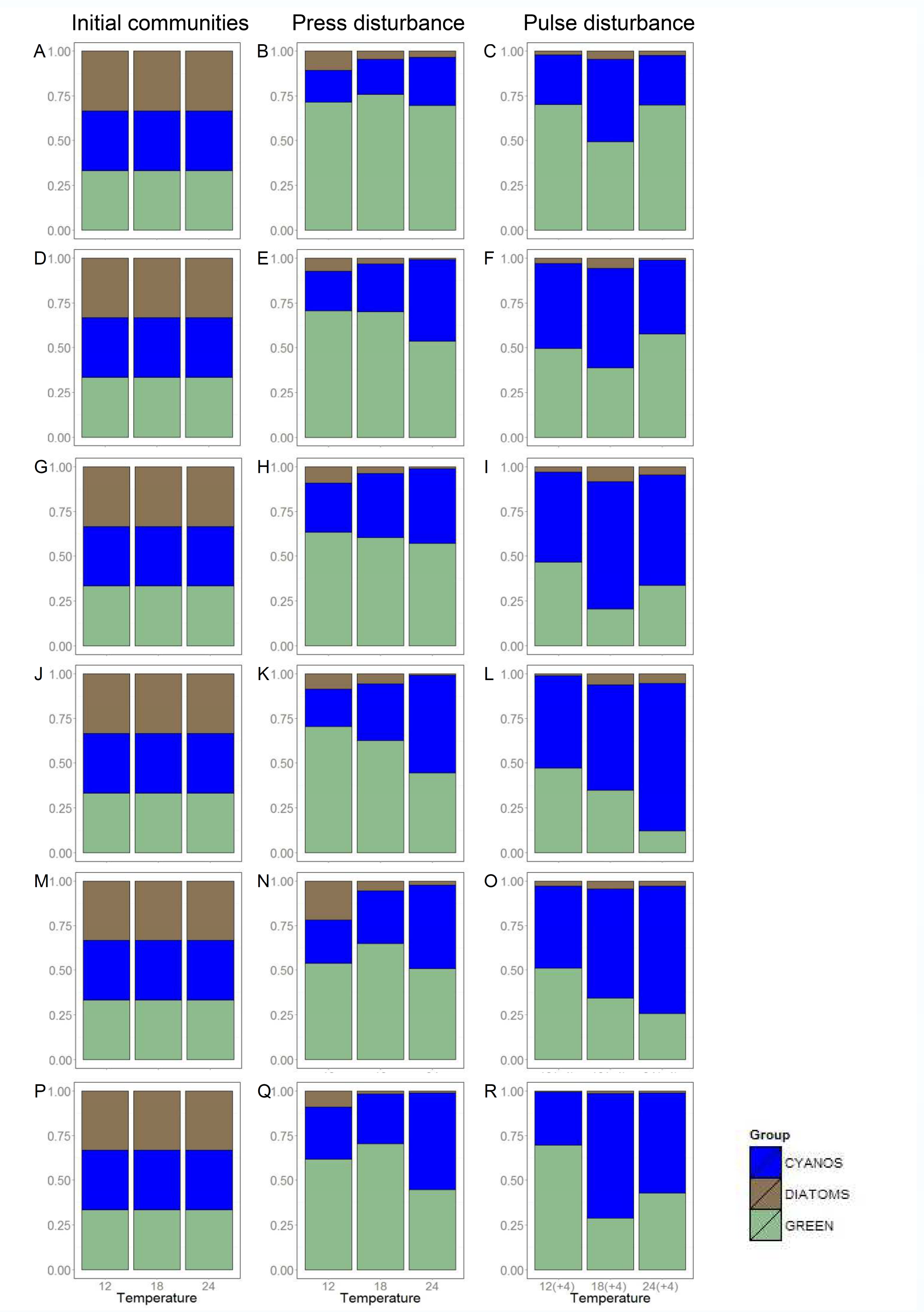

Online Resource 3: Community composition (relative biovolume) of artificial communities based on functional groups (Cyanobacteria, Diatoms, Green algae) for initial communities, communities after press disturbance and after peak disturbance. A-C Site1 (Danube River), D-F Site2 (Eberschütt Wasser). G-I Site3 (Hanselgrund), J-L Site4 (Kühworter Traverse), M-O Site5 (Schwarzes Loch), P-R Site6 (Schönauer Traverse).
